# Guidelines on DSE in patients selected for elective AAA repair need to reflect contemporary practice and evidence

**DOI:** 10.1186/s44156-026-00143-1

**Published:** 2026-11-09

**Authors:** A. Chaudhuri

**Affiliations:** Bedfordshire-Luton-Milton Keynes Vascular Centre, Bedfordshire Hospitals NHS Foundation Trust, Kempston Road, Bedford, MK42 9DJ UK

Dear Editors-in Chief,

It is noted that this guideline [1] by Steeds et al quotes the presence of an abdominal aortic aneurysm (AAA) >4cm in size as a relative contraindication to undertaking dobutamine stress echo (DSE) testing. There is no supporting citation for such a didactic statement, immediately rendering its validity questionable. This is particularly as DSE has been a part of the armamentarium of the pre-operative assessment pathways for the elective repair of AAA, with particular implications in the UK, where - like many other countries with advanced vascular surgical practice - vascular surgical centres (much like ours) have been centralised with benefits from multidisciplinary input including cardiology. Provocative testing such as DSE or cardiopulmonary exercise testing (CPET) have in our clinical practice unmasked coronary problems which have then been treated with priority, the issue around the application and predictive value of such tests are a different discussion [2–4].

The contraindication described thus flies in the face of both contemporary practice and in fact guidelines with greater reach, including those from the European Society of Vascular Surgery [5] which categorically indicates “~dobutamine stress echocardiography can be performed safely in patients scheduled for AAA repair” [6]. There are multiple other papers also indicating safety of DSE in patients with AAA [7], including clear conclusions that AAA rupture risk from DSE is low [8], which in fact specifically refutes the 4 cm threshold as a contraindication, but this has clearly not been included in either a relevant literature search and thus the references in this guideline. This is particularly relevant because AAA repair thresholds are at 55 mm in general, and even at 50 mm for female patients [9]. AAA <4 cm do not need treatment based on trial data, but the implication from this guideline is *that no AAA that is eligible for elective repair is eligible for DSE*! Similar urban myth around pulmonary function testing in patients with AAA for repair was also disproved [10].

It is notable this paper is from 2019, but it is vital to address how this is being interpreted and implemented even today, as it is being misinterpreted as an absolute contraindication by cardiologists not accustomed to multidisciplinary working linked to a centralised, regional vascular service. This contraindication in the guideline is thus inaccurate, and even years later leads to misleading multidisciplinary interactions with implications for delays in patient care pathways linked to elective AAA repair. Furthermore, given the uncited statement, it would seem reasonable to assume that such an unsubstantiated statement has possibly been made without the relevant multidisciplinary input including vascular anaesthetists/surgeons, and there should surely be clear merit in either amending such statements to reflect contemporary practice based on actual, contemporary data or removing it altogether, to prevent affecting patient care in this context.

## Data Availability

No datasets were generated or analysed during the current study.
